# Photo-induced Hall effect in metals

**DOI:** 10.1038/s41598-018-22776-x

**Published:** 2018-03-12

**Authors:** D. Li, A. Ruotolo

**Affiliations:** 1 Department of Materials Science and Engineering, City University of Hong Kong, Kowloon, Hong Kong SAR, China; 2 City University Shenzhen Research Institute, High-Tech Zone, Nanshan District, Shenzhen, 518057 China

## Abstract

The Hall effect in metals is too small to have practical applications. Instead, the same effect in semiconductors is the standard for magnetic field sensing. Yet, in semiconducting Hall-sensors, Joule heating severely compromises the linearity range. We here show that a Hall effect can be photo-induced in metals used for bias-free magnetic sensing. The system consists of a transparent metal that forms a Schottky contact to a semiconductor. Light reaching the interface results in the injection of charge from the space charge region. If a magnetic field is applied, a transverse, open-circuit voltage appears at the metal edges that is proportional to the field, as well as light intensity. The system shows sensitivities that are comparable to semiconducting Hall-sensors but no net current flows, therefore its performances are not affected by Joule heating.

## Introduction

**Figure 1:**
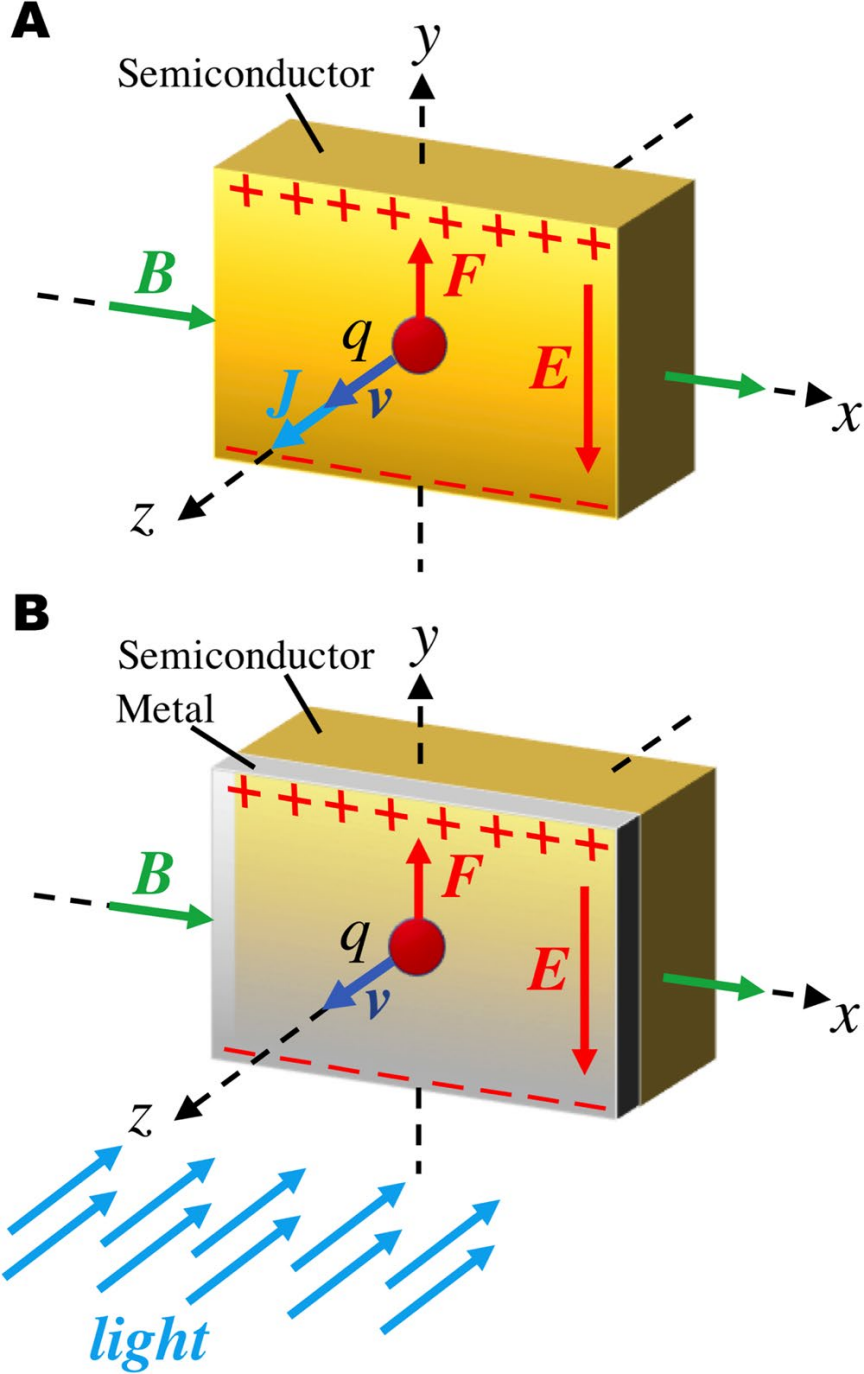
Current-biased Hall effect in semiconductors vs light-biased Hall effect in metals. (**A**) Charges drifting in a semiconductor are deflected by a perpendicular magnetic field and an electric field is produced that is perpendicular to both current and field directions; (**B**) Charges excited by light and diffusing in a metal are deflected by a magnetic field to produce an electric field that is perpendicular to both light and field directions.

The Hall-effect in semiconductors has been used for more than one century to measure the intensity of magnetic fields^1^. This is because the fundamental mechanism is simple, robust and linear. Charges *q* diffusing at a velocity ***v*** in a magnetic field ***B*** are subject to a magnetic force ***F*** = *q****v*** × ***B*** which results in the generation of an electric field ***E*** transverse to both ***v*** and ***B*** (see Fig. 1A). In photoconductive materials, the electric field can be enhanced by increasing the charge density through photo-excitation, an effect usually referred to as photo-Hall effect^2^.

While Hall sensors have been replaced by magnetoresistive sensors in digital systems^3,4^, they still retain two features which make them preferable to the latter in analog applications: they are intrinsically linear because they do not make use of magnetic materials and their sensitivity is directly proportional to the bias current ***J*** through the charge velocity ***v***. Yet, the latter of these features is more theoretical than practical. Commercial Hall sensors are engineered to work at a specific, and very low, bias current because Joule heating severely compromises their linearity. The Hall effect exists in metals, where the low resistance to electrical current would make Joule heating be negligible^5^. Yet, so would be the transverse voltage.

We engineered a system in which the Hall effect in a metal is greatly enhanced and Joule heating suppressed. The device recovers tunable sensitivity, without compromising linearity, by replacing current bias with light bias. Our system consists of an extended metal/semiconductor junction, where the metal is highly transparent to light and forms a Schottky interface with the semiconductor (see Fig. 1B). Light reaching the interface is the driving force to diffuse carriers across the bilayer. A magnetic field applied in the junction plane exerts a magnetic force on the carriers diffusing in the metal, which accumulate on opposite sides, according to their sign. This charge accumulation can be detected as an open-circuit voltage transverse to the metal. The voltage is proportional to the field strength, as well as to light intensity. At equilibrium, the net current is zero and no Joule heating is produces, regardless light intensity.

## Results

In order to demonstrate this effect, we deposited three metallic films, platinum (Pt), gold (Au) and aluminum (Al) with thickness *t* = 3 nm on 1 × 1 cm^2^ un-doped (100) silicon (Si) dice. These metals were chosen because they have progressively lower work-functions (*ϕ*_Pt_ = 5.9 eV, *ϕ*_Au_ = 5.5 eV and *ϕ*_al_ = 4.20 eV)^6^. While Pt and Au have work functions larger than that of Si (*ϕ*_Si_ = 4.6 eV), which favors formation of Schottky barriers, the work function of Al is lower than that of Si, therefore Al tends to form ohmic contacts to Si. A Si dye and a sample consisting of Pt with the same thickness deposited on insulating sapphire (α-Al_2_O_3_) were used as references. The magnetic field was generated by using an electromagnet. The voltage was measured by connecting the midpoints of two opposite edges of the metallic film to a voltmeter. A light source consisting of a bulb lamp and a manual shutter were employed. The bulb lamp emitted unpolarized light, which excludes possible contributions to our measurements from spin-injection Hall and photo-voltaic Hall effects^7,8^. The light had a measured spectrum that covered all and only the visible range from *λ* = 450 nm (violet) to *λ* = 750 nm (red).

It must be understood that ultra-thin metallic films are highly transparent to light^9^. Light penetration depth is wavelength dependent: *δ* = √(*ρλ*/π*μc*), where *ρ* is the resistivity of the metal, *λ* is the wavelength, *c* is the speed of light in vacuum and *μ* is the magnetic permeability. For instance, for our Pt, with an as-measured resistivity of *ρ*_Pt_ = 2.1 × 10^−6^ Ω m, *δ* = 2.8 nm for *λ* = 450 nm (violet) and *δ* = 3.6 nm for *λ* = 750 nm (red). Therefore, visible light can reach the interface for a Pt film of *t* = 3 nm. Similarly, the resistance offered by the film of size *l* = w = 1 cm *R* = *ρl*/*wt* ~ 1 kΩ. Therefore, a significant difference of potential can fall between its edges.

**Figure 2:**
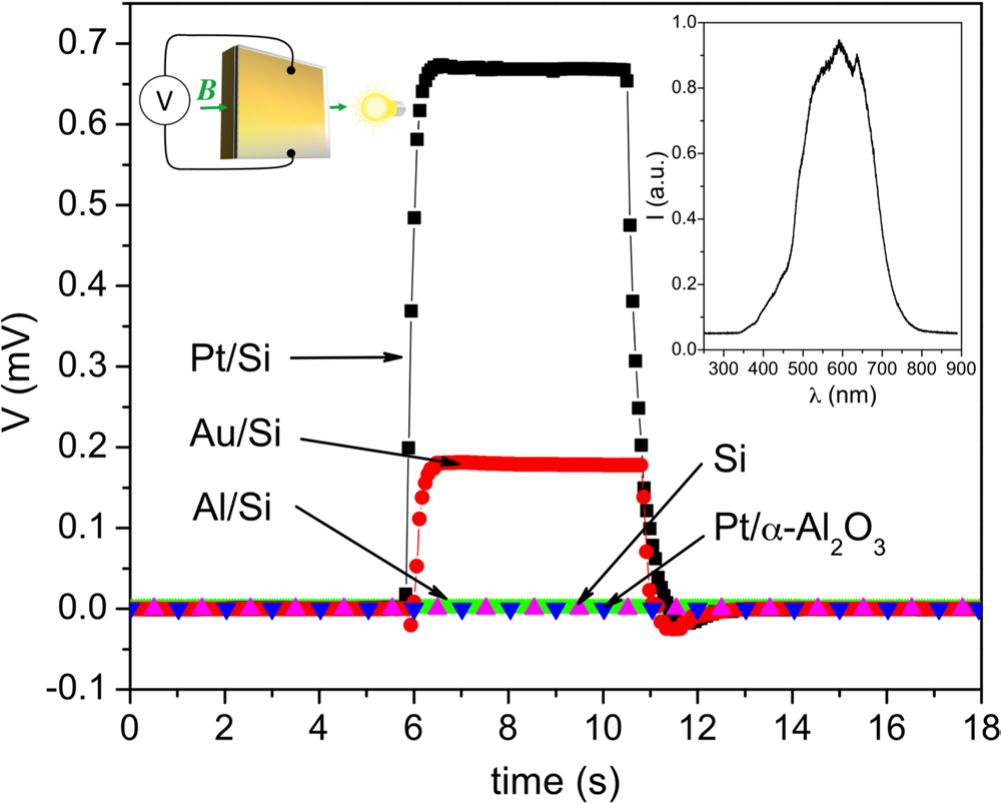
Photo-excitation of transverse electric field in perpendicular magnetic field. Voltage *vs* time elicited when exposing the samples to light with intensity *I* = 1 W and *B* = 0.5 T. The upper right inset shows the spectrum of the light.

Figure 2 shows the voltage *vs* time elicited when exposing the samples to a light with intensity *I* = 1 W and magnetic field *B* = 0.5 T. One can see that no voltage appears when a Si dye is used, which excludes that the effect is due to photo-Hall effect in the semiconductor^2^. Similarly, no signal was detected on a Pt/sapphire chip, which excludes that the voltage is due to excitation of electrons in the Pt. One can also see that the effect diminishes with decreasing work-function, which suggests that the establishment of a Schottky barrier is a necessary condition for the effect to exist and that light is absorbed in the space-charge layer of the junction.

**Figure 3:**
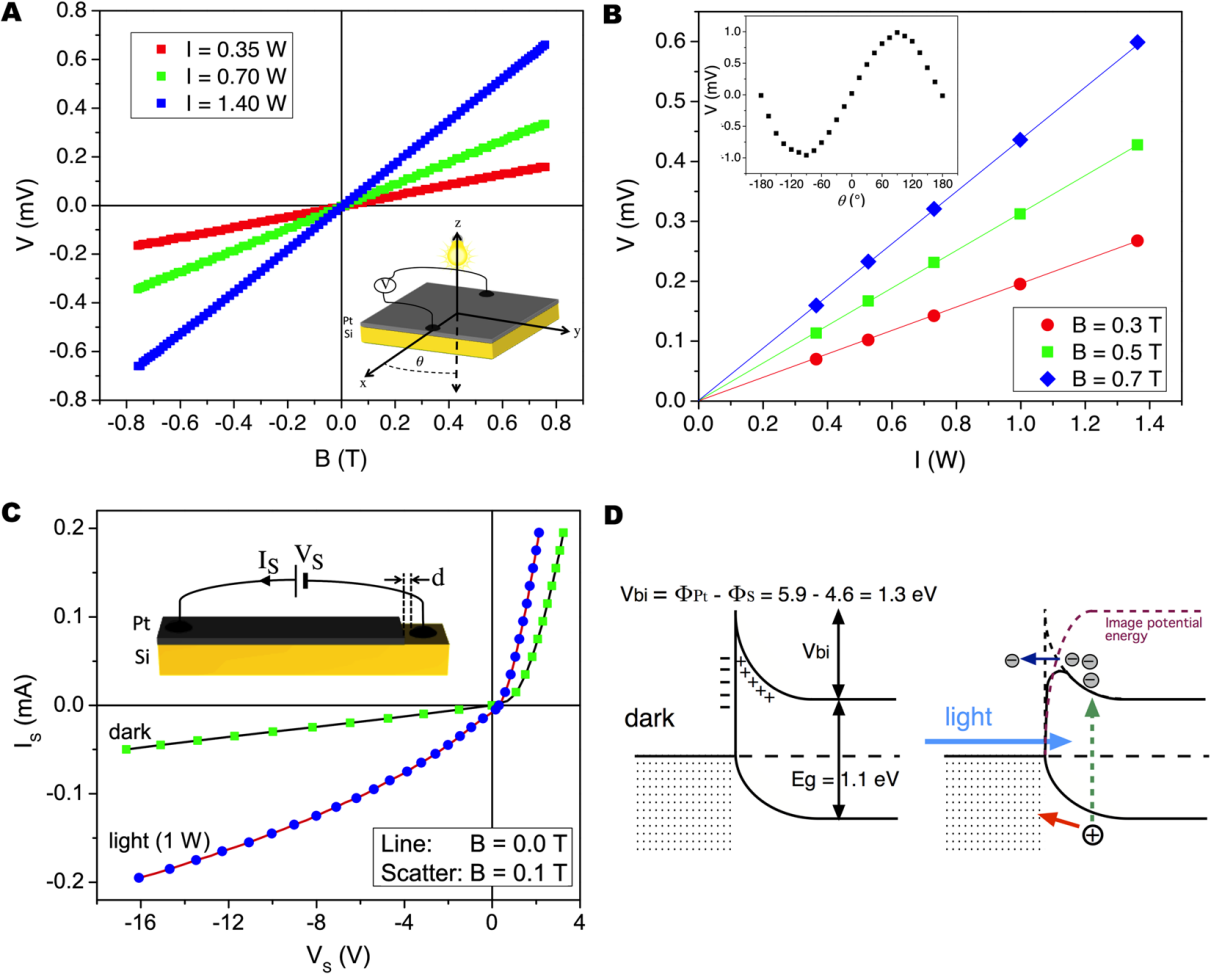
Photo-induced Hall effect in platinum and its origin. (**A**) Voltage *vs* magnetic field at constant light intensities for a Pt/Si chip. The lower-right inset shows the measurement setup. (**B**) Voltage *vs* light intensity at constant magnetic fields of the same sample. The upper-left inset shows the normalized voltage *vs* in-plane angle at a fixed field strength and light intensity; (**C**) Current-voltage characteristics of a Pt/Si interface in the dark and in the light. Scatter curves show the same characteristics when a magnetic field is applied in the sample plane. Inset shows the measurement configuration; (**D**) Sketch of the photo-induced excitation of charges and lowering of the barrier due to image forces. *V*_*bi*_ is the theoretically calculated built-in potential.

For Pt and Au, the effect was found to be strongly linear with both magnetic field strength and light intensity. In Fig. 3A we show the detected voltage as a function of the field strength for different light intensities. The voltage was proportional to the magnetic field strength for values up to the maximum we can apply with our electromagnet (~0.8 T). Similarly, in Fig. 3B, the voltage increased linearly with light intensity for fixed values of magnetic field. In order to understand whether the effect was due to magnetic forces on charges diffusing in the *z*-direction, we measured the voltage as a function of the angle *θ* between the field and the voltage direction by rotating the sample holder in the *x-y* plane (Fig. 3B inset). As expected, the voltage showed a sinusoidal dependence with *θ*.

Si has a band-gap *E*_*g*_ = 1.1 eV, corresponding to a wavelength λ = 1127 nm, and therefore can absorb light in the visible range. Yet, charge photo-excited in the Si and simply diffusing in the metal would produce a transverse, open-circuit voltage no matter the metal employed. Besides, this voltage should be very small, because so is the Hall effect in a metal. In order to understand why the effect appears only when a metal with high work-function is employed, we deposited Pt on a Si slab after masking part of the chip with a shadow mask (see Fig. 3C). The current-voltage (*I*_*S*_/*V*_*S*_) characteristic across the Pt/Si interface was measured by placing the electric contact on the Si as close as possible to the edge of the Pt. Despite the fact that the gap *d* between the Pt edge and the contact to the un-doped Si provides a significant series resistance, which hinders us from estimating the built-in electric field, one can well appreciate that Pt forms a Schottky contact to the semiconductor. The *I*_*S*_*/V*_*S*_ was found to be strongly dependent on the light and independent on the magnetic field. On the other hand, the magnetic field cannot change the charge distribution in the *x-y* plane of neither the space-charge region nor the semiconductor, because magnetic forces only act on moving charges in the *z*-direction for an in-plane field.

Surprisingly, light does not produce either a rigid downshift of the *I*_*S*_*/V*_*S*_ along the current-axis, as in a photo-diode, or a rigid shift along the voltage-axis, which would indicate a shift of the Fermi levels equivalent to the application of an external electric field, but rather an increase of both direct and reverse current, as if light lowered the barrier height, hence driving the contact from Schottky to quasi-ohmic (see Fig. 3C). A lowering of the barrier height of a Schottky contact or, equivalently, an unusual increase of the leakage current in reverse bias, has well been explained and modeled by the theory of the image forces^10^.

## Discussion

The experimental evidences lead us to draw the following scenario, depicted in Fig. 3D. Metals with high work-functions form a Schottky contact with the semiconductor. As a Schottky contact is made, electrons in the conduction band of the semiconductor have a higher energy than electrons in the metal. As a consequence, they tunnel into the metal leaving uncompensated positive charge in the semiconductor. At equilibrium, the built-in potential *V*_*bi*_ prevents additional electrons to migrate. This equilibrium corresponds to the alignment of the Fermi levels. When light is shed, if the light penetration depth through the metal is longer than the film thickness, photons can be absorbed and electron-hole pairs are generated^11^. A hole can readily be neutralized by an electron transferred from the metal but an electron cannot easily overcome the barrier or flow through the highly-resistive, intrinsic semiconductor. Yet, an additional electron in the space charge region results in a lowering of the barrier height that can be modeled with the theory of image forces^10^. An electron a distance *x* from the interface induces a positive mirror image charge in the metal at distance *−x* and therefore a negative potential energy that opposes the positive potential due to the Schottky barrier. As the negative potential arising from the Coulomb force between electron and image charge decreases with the distance *x* from the interface, this results in a rounding of the net potential barrier as depicted in Fig. 3D. If the semiconductor is highly resistive and photo-exited electrons cannot easily be discharged through it, which is the case here, the lowering of the Schottky barrier becomes significant, as reveled by the significant increase of the leakage current in reverse biased in Fig. 3C. In open-circuit conditions, as negative charge builds up, electrons with higher energy can cross the barrier. Electrons must acquire a substantial velocity for a large Hall voltage to appear on the edges of the metal. This velocity can easily be estimated by imposing equilibrium between Lorenz forces and electric forces in the metal: *evB* = *eV/w*, where *e* is the electron charge and *w* is the distance between the contacts (see inset Fig. 1A), in our case *w* = 1 cm. We estimated an average velocity *v* = 62 m/s per Watt. This velocity is much smaller than the saturation velocity in silicon, which is 10,000 m/s for electrons and 6,000 for holes^12^.

The immediate foreseeable application for the system we have presented here is sensing of magnetic fields. Let us therefore compare our prototype device with commercial Hall sensors. First, standard Hall sensors require four contacts (two for the bias current, two for the measured voltage) whereas our sensor requires only two contacts. Standard Hall sensors offer a sensitivity of ~1 mV/T when biased with 0.1 mA^13^. The non-optimized system in Fig. 3 can yield the same sensitivity for a light intensity of ~1 W. In the former, attempts to bias at higher currents to increase sensitivity would make the linearity-error increase significantly if no heat sink is used. Our sensor has a linearity-error that is independent on sensitivity because no Joule heating is produced. If cold light sources, such as white LEDs are employed, the temperature of the sensor would be unaffected by the light. Let us finally point out that the sensitivity of our system can be greatly increased in different ways. First, doping Si would increase the diffusion current, and therefore the sensitivity. On the other hand, it would reduce the Schottky barrier width (linearization of the *I*_*S*_/*V*_*S*_ because of direct or thermally-assisted tunneling)^14,15^ and therefore reduce the sensitivity. The trade-off must be found by carrying out a systematic study with doping concentration. Another way to increase the sensitivity is to reduce the thickness of the metallic film. A thinner film would be more transparent and more resistive, therefore the benefits would be two-fold. Replacing Si with a direct band-gap semiconductor, such as GaAs, should also increase sensitivity.

## Materials and Methods

The silicon wafers used in the experiment were purchased from Siltronix (France). They were single-side polished, (100)-oriented, intrinsic silicon wafers with a resistivity greater than 10,000 Ωcm. Metal targets were 99.99% pure and purchased from Kurt J. Lesker. Metallic films were deposited on as-received silicon by d.c. magnetron sputtering at room temperature and in Argon atmosphere of *P*_*Ar*_ = 0.5 Pa. Before deposition, the wafers were diced in chips of 1 × 1 cm^2^ for photo-Hall measurements and slabs of 1 × 0.3 cm^2^ for current-voltage characterization. The slabs were partially masked to access the silicon surface. Electric contacts were made by aluminum wire bonding for the chips and epoxy for the slabs.

The magnetic field was generated by a water-cooled electromagnet. The light source was an optical fiber illuminator Helmut Hund GmbH type FLQ 85E. No optical fiber was connected. The light intensity was measured by using a Newport digital power meter after removing all the filters from the sensor and multiplying by the ratio between the sample area and sensor area. Open circuit voltage and current-voltage characteristics were measured by using Keithley multimeters.

## References

[1] Hall E (1879). On a New Action of the Magnet on Electric Currents, Am. J. Math..

[2] Tyler WW, Woodbury HH (1956). Scattering of Carriers from Doubly Charged Impurity Sites in Germanium. Phys. Rev..

[3] Baibich MN (1988). Giant Magnetoresistance of (001)Fe/(001)Cr Magnetic Superlattices. Phys. Rev. Lett..

[4] Binasch G, Grunberg P, Saurenbach F, Zinn W (1989). Enhanced magnetoresistance in layered magnetic structures with antiferromagnetic interlayer exchange. Phys. Rev. B.

[5] Hurd, C. M. *The Hall Effect in Metals and Alloys* (Plenum Press, New York, 1972).

[6] Rivìere, J. C. Work Function: Measurements and Results, edited by M. Green, Solid State Surface Science, Vol. **1** (Decker, New York, 1969).

[7] Wunderlich J (2009). Spin-injection Hall effect in a planar photovoltaic cell. Nat. Phys..

[8] Oka T, Aoki H (2009). Photovoltaic Hall effect in graphene. Phys. Rev. B.

[9] Heller A, Aspnes DE, Porter JD, Sheng TT, Vadimsky RG (1985). Transparent Metals: Preparation and Characterization of Light-Transmitting Platinum Films. J. Phys. Chem..

[10] Rhoderick, E. H. & Williams, R. H. *Metal-semiconductor contacts*, 2^nd^ ed. Oxford University Press, 1988.

[11] Quimby, R. S. *Photonics and Lasers: An Introduction*, (Wiley-Interscience, New Jersey, 2006).

[12] Sinitsky D, Assaderaghi F, Hu C, Bokor J (1997). High field hole velocity and velocity overshoot in silicon inversion layers. IEEE Electron Device Lett..

[13] Popovic, R. S. *Hall-effect devices, Sens. and Actuators* **17**, 39 (1989).

[14] Padovani FA, Stratton R (1966). Field and thermoionic-field emission in Schottky barriers. Solid State Electron..

[15] Chang CY, Sze SM (1970). Carrier transport across metal-semiconductor barriers. Solid State Electron..

